# Optimal Timing for Neonatal Hearing Screening in Well-Babies

**DOI:** 10.3390/ijns12010007

**Published:** 2026-02-15

**Authors:** Lisanne Vonk, Paula van Dommelen, Iris Eekhout, Noëlle N. Uilenburg, Paul H. Verkerk, Catharina (Kitty) P. B. van der Ploeg

**Affiliations:** 1 Child Health, Netherlands Organisation for Applied Scientific Research (TNO), P.O. Box 3005, 2301 DA Leiden, The Netherlands; 2 Dutch Foundation for the Deaf and Hard of Hearing Child (NSDSK), Lutmastraat 167, 1073 GX Amsterdam, The Netherlands

**Keywords:** universal neonatal hearing screening, otoacoustic emissions, well babies, permanent congenital hearing impairment, screening age, false positive rate

## Abstract

In The Netherlands, preventive child healthcare (PCHC) has been carrying out neonatal hearing screening in well-babies since 2006. The aim of this study was to examine the relationship between the age of newborns and the false positive referral rate of the first hearing screening using a transient evoked otoacoustic emission (OAE) test, to identify the most efficient timing for OAE screening. Additionally, we investigated the relationship between the type of OAE screening device (Echoscreen (ES)I/II versus ESIII) and the referral rate during the first screening. We used data from the Dutch universal well-baby neonatal hearing screening programme by PCHC between 2013 and 2023. Multilevel logistic regression analyses were performed to estimate the probability of a referral in 2023 for newborns screened in 2022 and 2023. We included a total of 1,650,506 newborns for 2013–2022 and 323,194 newborns for 2022–2023. The lowest false positive referral rates were found between days five and thirteen, ranging from 3.3 to 3.9%. ESIII significantly increased the probability of a referral compared to ESI/II (odds ratio = 1.84, 95% confidence interval = 1.65–2.06). In conclusion, the timing of neonatal hearing screening significantly impacts the false positive referral rate. Furthermore, the likelihood of a referral is significantly higher when using the ESIII compared to the ESI/II.

## 1. Introduction

Hearing impairment is among the most prevalent sensory disorders in children globally [1]. In The Netherlands, the prevalence of unilateral and bilateral hearing impairment of ≥40 dB is 1.23 per 1000 well-babies [2]. This prevalence rate is in line with those in other studies [3]. Neonatal hearing screening is crucial, as timely treatment can lead to better health outcomes. Yoshinaga-Itano et al. [4,5] found that infants with bilateral hearing impairment who receive early intervention within six months after birth typically show better language and speech development. Therefore, developed countries worldwide perform universal neonatal hearing screening [6]. Currently, there are two primary methods for neonatal hearing screening, one based on otoacoustic emissions (OAEs) and the other on automated auditory brainstem responses (AABRs) [7]. The OAE method is widely implemented due to its speed and costs [8,9,10]. However, like any screening test, it may also give false positive results. A common reason for a referral is the presence of residual amniotic fluid in the middle ear [11]. The timing of the hearing screening after birth is crucial, as it impacts the false positive referral rate, yet little is known about the optimal timing [10]. A study in India demonstrated that screening on day five after birth is more optimal compared to earlier days [10]. The false positive rate was 2% on day five, compared to 92% on day one. Nevertheless, the study did not examine effects beyond day five. Moreover, the authors concluded that this study should be replicated across varied demographic and geographical settings as neonatal care practices vary.

In The Netherlands, preventive child healthcare (PCHC) has been carrying out neonatal hearing screening in well-baby newborns since 2006 [12]. Approximately 75% of screenings are performed during a home visit, in combination with neonatal blood spot screening via a heel prick. These home visits are part of standard postnatal care in The Netherlands. The heel prick is recommended as soon as possible from 72 h after birth. This is a compromise, taking into account that for some conditions neonatal blood spot screening as early as possible is best, while for others this can best be performed several days after birth. Thus, if performed simultaneously, the hearing screening should also be performed around that time. However, it was found in 2007 that newborns screened on day three had a higher false positive rate on hearing screening by the OAE method than those screened on day four [12]. This resulted in the decision by the Dutch Centre of Population screening to perform the combined screening from 96 h after birth. With fewer false positives, screening is performed more efficiently, saving both time and financial resources. Additionally, false positives can cause unnecessary anxiety among parents [13]. Since 2007, many newborns have had hearing screening, providing the opportunity to re-evaluate the relationship between the age at screening and the false positive referral rate. This is relevant for optimizing neonatal hearing screening with the OAE method both nationally and internationally.

Besides the timing of screening, the type of screening device may also influence the referral rate. Annual monitors of the neonatal hearing screening programme by PCHC showed that the referral rate after the first OAE screening test has been rising recently, from 4.4–4.8% in 2017–21 to 5.1% in 2022 and 5.4% in 2023, while the prevalence of hearing impairment has not increased [14]. We hypothesized that this increase can partly be attributed to the transition from the OAE screening device Echoscreen (ES) I or II to the newer ESIII, which has been increasingly adopted since 2021.

We aimed to examine the relationship between the age of newborns at the first hearing screening using the transient evoked OAE test and the false positive referral rate, to identify the most efficient timing for screening. Additionally, we investigated the relationship between the type of OAE screening device (ESI/II versus ESIII) and the referral rate during the first screening.

## 2. Materials and Methods

### 2.1. National Neonatal Hearing Screening Programme

In The Netherlands, PCHC has been performing neonatal hearing screening in newborns since 2006 [12]. Newborns admitted to neonatal intensive care units (NICUs) (about 2% of all newborns) are screened through a separate programme, due to their higher risk of hearing impairment, and are therefore not included in this study [15]. The aim of the neonatal hearing screening programme is to identify children with permanent congenital hearing impairment of a minimum of 40 dB in one or both ears, so that an appropriate intervention may be provided before the age of six months. The neonatal hearing screening programme has been described in detail elsewhere [12,16]. In short, regional PCHC organizations screen newborns in up to three sessions, using transient evoked OAE screening (Echoscreen) in the first two sessions and AABR in the third session. The ES screening device is explained in more detail below. Each of the three screening sessions allows for preferably a maximum of three tests per ear, until a ‘pass’ result is achieved. If all tests on one or both ears only give refers or invalid results but no ‘pass’, the overall screening result of the session is positive, which makes the child eligible for referral to the next session. After the third session, children with a recurring refer or invalid result are referred to a Speech and Hearing Centre for diagnostic investigations. In The Netherlands, most newborns are born at home or in a birth clinic, with discharge usually occurring shortly after birth. Hearing screening is most often performed at home on day four after birth or soon thereafter. However, PCHC organizations also have the option to conduct the screening at a well-baby clinic (WBC) [16]. Such clinic screenings occur around three weeks of age. The hearing screening is performed by trained nurses from the PCHC [16]. Training covers, for example, performing the screening and using the screening device. Test results, such as the date, duration, and outcome (’pass’ or ’refer’) of all screening sessions, as well as child variables, are automatically stored in a centralized software system supporting the screening programme throughout the country. This database is managed by the Dutch Foundation for the Deaf and Hard of Hearing Child (NSDSK), on behalf of the PCHC organizations.

### 2.2. Echoscreen

At the introduction of the neonatal hearing screening programme in The Netherlands, the ESI was used for screening with OAEs. In 2006, the ES was acquired by another company and the ESI was updated to the ESII. There were no differences between the ESI and ESII devices except for some external features. In October 2014, the end of sale of the ESII was announced and the ESIII was introduced. Introduction of the ESIII into daily screening practice occurred gradually from 2016 onwards. Screeners reported having more difficulties with inserting the probe of the ESIII, and monitoring showed an increase in referral rate over the years that the ESIII was further implemented. The end of sale of the ESIII was announced by the end of 2022, with seven years of service and maintenance remaining. Although the ESI, ESII, and ESIII can no longer be purchased, they are still in use within the screening program. Note that ESI, II and III refer to the different versions of the ES device and are not related to the different screening sessions.

### 2.3. Study Population and Datasets

For this study, two anonymous datasets from participants of the national neonatal hearing screening programme by the PCHC born between 2013 and 2023 were retrieved from the NSDSK database.

The first dataset was used to determine the relationship between age at the first screening session and false positive referral rates, i.e., the percentage of newborns who incorrectly did not pass the first test. Whether newborns had a false positive result in the first test was based on results from subsequent screening sessions. This assessment was made before a newborn was referred to the Speech and Hearing Centre, since diagnostic outcomes were not available in the dataset. The first dataset included newborns born between 2013 and 2022. Data from newborns with a first screening with the AABR were not included in this dataset. A subset of the first dataset (birth years 2020–2022) was used for a preliminary analysis of false positive referral rates by device type (ESI/II and ESIII). The dataset contained per newborn the results for the left and right ear in the first hearing session with OAEs (pass, refer, failed measurement, or empty), as well as the day of the first screening (with the day of birth as day 0), the type of PCHC organization (screening at home, often combined with the heel prick, or at the well-baby clinic), the ID number of the PCHC organization, the type of Echoscreen device (I/II (combined) or III), and the year of birth. Also, incomplete data on prematurity (‘no’ or ‘yes’) were provided: this was ‘yes’ if the due date was at least three weeks after the birth date. However, this was only known if the newborn was screened at 14 days after birth or later.

The second dataset included only data from newborns born in 2022 and 2023 and was used for more detailed analyses regarding the screening device. This dataset was similar to the first dataset but included more recent data.

### 2.4. Exclusion Criteria

To investigate the relationship between age at the first screening session and the false positive rate, we aimed to exclude true positive cases, i.e., children with a hearing impairment of 40 dB or more in one or both ears. However, diagnoses of the children that were referred to a Speech and Hearing Centre after completion of the complete screening of three sessions were stored in a separate database, which was not accessible for this study. Therefore, we could not exclude true positives only but instead excluded all children eligible for referral to the Speech and Hearing Centre after three screening sessions, i.e., all newborns with positive first, second, and third screening sessions. Thus, we likely excluded all true positives but also a small part of the false positives. This unwanted exclusion of false positive cases concerned an estimated 0.2% of the total population, as yearly monitors show that around 0.3% of all children are referred to the Speech and Hearing Centre after the complete screening of three sessions, and about two-thirds of these referred newborns are false positives [14]. Additionally, newborns registered as premature were excluded, since premature newborns form a special group that is often screened at an older age. Premature newborns were defined as those born before 37 weeks of gestation. Newborns whose screening was registered on day 0, on day 1, or beyond six months were excluded, as such timings are likely due to registration errors. According to the neonatal hearing screening protocol, newborns may be screened from 96 h after birth up to the age of three months. Therefore, a registration error in the date of screening or date of birth may have occurred in these cases. For the second dataset, we excluded children screened with the AABR, since our study focused on the first OAE screening with the ES. We did not exclude true positive cases or children that were referred to the Speech and Hearing Centre in these analyses. Thus, in this analysis, effects on the referral rate of the first OAE screening were investigated instead of effects on the false positive rate.

### 2.5. Statistical Analyses

For the first screening session, the OAE results of the left and right ear were combined into one result, where only newborns with a sufficient result (‘pass’) in both ears were considered ‘no referral’. All other combinations of results were classified as ‘referral’. Referral rates were calculated by age at screening. Age at screening was calculated in days. The day of birth was defined as day 0.

For the first research question, we created a graph illustrating the association between the age at the first hearing screening and the false positive referral rate. Additionally, we demonstrated the relationship between the age at the first hearing screening with the OAE method and the false positive referral rate, stratified by screening device using data from 2020–2022. Both analyses included the 95% confidence intervals (CIs).

For our second research question, we used SPSS v29 [17] to perform multilevel logistic regression analyses for the probability of a referral in 2023. To account for the nested structure of the data, we used a two-level model for the analyses, where newborns were nested within PCHC organizations. This approach was necessary because the probability of a referral may vary between organizations. The following variables were included in the regression model: the type of OAE device (ESI/II or ESIII), age at first screening, type of PCHC organization (either screening at home combining hearing screening with a heel prick, or screening at the well-baby clinic), and use of the ESIII in 2022 as a measure of experience with the device within each organization (as a percentage of all first OAE screenings). Furthermore, an interaction term between experience and device type was included. This tested whether the level of experience with the ESIII in the organization in the previous year affected the probability of a referral with the ESIII. Finally, an interaction term between age and device type was included. This tested whether the probability of a referral with the ESIII varied for the different age groups. Data from the ESI and ESII were combined, as the only reason for the change in device number was the transition to another producer and some external features.

## 3. Results

### 3.1. Relation Between Age and False Positive Referral Rate at First OAE Screening

#### 3.1.1. Excluded Newborns

Dataset 1 contained data from 1,663,646 newborns screened between 2013 and 2022. Of these, 12,863 (0.8%) were registered as premature and were excluded. Also, term children registered as being screened on the day of birth (*N* = 122), on day one (*N* = 50), or over six months after birth (*N* = 105) were excluded (0.02% of the 1,650,783 term newborns).

#### 3.1.2. Age at First OAE Test and False Positive Referral Rate

**Figure 1:**
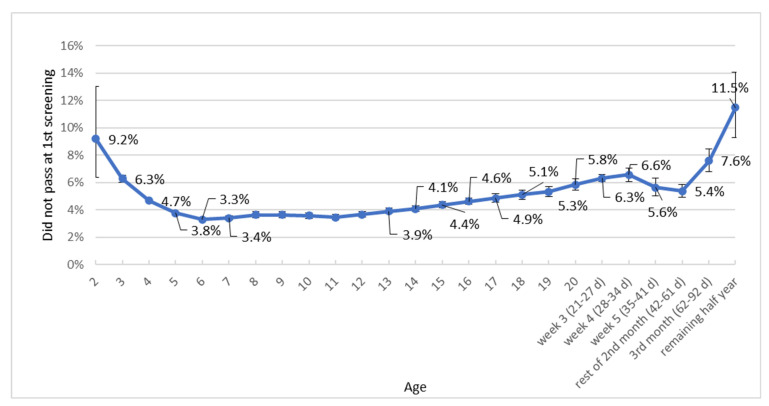
Relationship between age at first OAE hearing session and false positive referral rate (*N* = 1,650,506). Note: Bars: 95% confidence intervals.

Figure 1 is based on dataset 1 and presents the relationship between age at the first OAE hearing screening session and the false positive referral rate for all years combined (*N* = 1,650,506). The lowest false positive referral rates were observed between days five and 13, ranging from 3.3% to 3.9%. At younger ages, these rates were higher (6.3% on day three, 4.7% on day four). After day thirteen, the rates increased again, reaching 5.8% on day 20, 6.3% in week three, and 6.6% in week four, then unexpectedly decreasing to 5.6% in week five and stabilizing around 5.4% during the remainder of the second month.

#### 3.1.3. Type of Echoscreen

**Figure 2:**
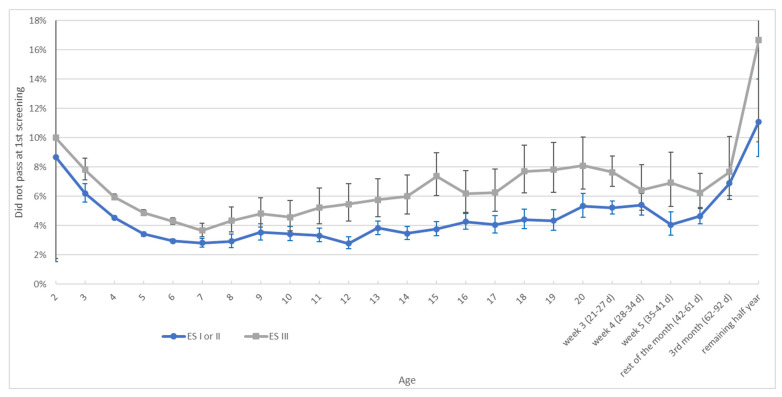
Age in days at first OAE hearing session and false positive referral rate by screening device (*N* = 495,669: 331,550 for ESI/II and 164,119 for ESIII). Note: Data from 2020 to 2022 combined. Bars: 95% confidence intervals.

In recent years, the ESI and ESII have increasingly been replaced by the ESIII. From 2020 to 2022, the ESI/II and ESIII had average false positive referral rates of 3.9% and 5.4% respectively (Figure 2). At every age, the ESIII has a higher rate than the ESI/II. The percentage of newborns screened with the ESIII has been increasing over time, with 25.2% in 2020, 32.5% in 2021, and 41.8% in 2022.

### 3.2. Relationship Between Type of OAE Screening Device and Referral Rate

In total, dataset 2 contained data from 323,507 newborns, with 163,429 newborns born in 2022 and 160,078 in 2023. Data from newborns screened by the AABR method in the first session were excluded (147 newborns in 2022 (0.1%); 166 newborns in 2023 (0.1%)), leaving data from 323,194 newborns screened by OAE testing (ESI/II or III).

#### Type of OAE Screening Device

Referral rates were consistently higher for the ESIII compared to the ESI/II in both 2022 and 2023 (Table 1). While the referral rates for the ESI/II increased slightly in 2023 compared to 2022, the referral rate for the ESIII remained stable across both years.

The results of the multilevel logistic regression analysis for the probability of a referral at the first OAE screening in 2023 are shown in Table 2. Based on the results of the first research question, we included age in three categories: till day four, day five to thirteen (reference), and ≥day fourteen. Compared to the ESI/II, the ESIII significantly increased the probability of a referral (odds ratio (OR) 1.84, 95% CI 1.65–2.06). This number applies to the reference group of variables, which is at the optimal age of five to thirteen days, at home, and without any experience with ESIII within the organization (0% ESIII use in 2022). With maximum experience (100% ESIII use in 2022, see experience with interaction term), the OR decreased from 1.84 to 1.25 (namely 0.63*1.09*1.84). Therefore, with full experience using the ESIII in 2022, the ESIII gave 1.25 times more referrals compared to the ESI/II.

The model also confirms that the probability of a referral depends on age at screening. Both newborns who were screened earlier than day five and those who were screened later than day 13 had a higher probability of a referral (ORs 1.28 and 2.05 respectively). The other variables had little effect. For example, whether screening is done by a PCHC organization screening at home or at the well-baby clinic did not affect the probability of a referral when adjusted for the other variables presented in Table 2.

An interaction effect between age and device type was also observed. The increased referral probability associated with the ESIII was less pronounced in newborns screened later than thirteen days compared to those within the optimal age range (5–13 days) (OR 0.83). At this higher age, the probability of a referral with the ESIII was 1.53 times higher than with the ESI/II (namely 0.83*1.84), while at the age of five to thirteen days it was 1.84 times higher (Table 3). Although the ratio varied with the age group, Table 3 shows that referral rates for ESIII were consistently higher than those for the ESI/II across all age groups.

## 4. Discussion

The aim of this study was to examine the relationship between the age of newborns during the first hearing screening with OAE testing and the false positive referral rate, i.e., the percentage of newborns who incorrectly did not pass the first test, to identify the most efficient timing for screening. Additionally, we investigated the relation between the type of OAE screening device (ESI/II versus ESIII) and the referral rate during the first screening.

Our findings based on 1.6 million well-baby newborns showed that the association between age at the first screening session with OAE testing and the false positive referral rate is comparable for the years 2013–2022 to the pattern found in 2007 with data from 2006 [12]. The lowest positive referral rate was observed between days five and thirteen (3.3–3.9%; screening at home or at the well-baby clinic combined). Towards younger ages, an increase is visible (6.3% on day three, 4.7% on day four, and 3.8% on day five). After day thirteen, an increase is also visible to 5.8% on day 20, 6.3% in week three, and 6.6% in week four.

Our results are in agreement with the findings of Kumar et al. [10]. They found that day five was significantly better for performing the hearing screening compared to earlier days. However, as the study of Kumar et al. focused on hearing screening during the first five days after birth, no conclusions can be drawn for later time periods. Preventing false positive first screening results reduces unneeded further hearing screenings and diagnostics, thus reducing financial and time costs for healthcare professionals and parents, and preventing needless worry among parents [13]. Therefore, it is important to take the timing of hearing screening into account.

When neonatal hearing screening is combined with neonatal blood spot screening, through a heel prick preferably at 72 h after birth, there is particular interest in the neonatal hearing screening results on days three and four. For hearing screening, it is less favourable to screen newborns on day three: the false positive referral percentage then is 6.3%, while on day four it is 4.7%. Therefore, more newborns would need a second test due to a false positive result if screened on day three. When combined heel prick and hearing screening was introduced in The Netherlands, it was decided in 2007 that this should be done from day four after birth due to the higher referral percentage for hearing sessions on day three. From the perspective of the hearing screening, this approach remains justified.

The false positive referral rates found in 2013–2022 were lower than those in 2006. In 2006, the figures were 7.5% on day three (1.2% higher than now) and 6.0% on day four (1.3% higher than now) [12]. Unfortunately, this gain has recently been lost after the transition to the ESIII. The percentage of newborns screened with the ESIII has increased over time (from 25.2% in 2020 to 41.8% in 2022). Our findings from 2020 to 2022 show that the ESI/II had a false positive referral percentage of 3.9%, while the ESIII had 5.4%. This is in line with our findings from the multilevel analysis: it appears that the likelihood of a referral is significantly higher when using the ESIII compared to the ESI/II, especially if a PCHC organization has little experience with the ESIII. If there is already considerable experience with the ESIII, the likelihood of a referral with the ESIII decreases but still remains higher compared to that using the ESI/II, despite the high level of automation of these devices. Although the ESI, ESII, and ESIII devices are still in use within the screening programme, they are no longer available for purchase. Consequently, it is not feasible to give recommendations regarding the use of these screening devices. Nevertheless, our findings demonstrate that the screening device used has a significant influence on the false positive referral rate. Therefore, we strongly recommend monitoring false positive rates and other test outcomes in daily practice in a pilot phase before introducing new equipment on a large scale in a screening programme.

### Strengths and Limitations

This study had several strengths. We obtained data from all newborns who underwent neonatal hearing screening conducted by the PCHC, resulting in a dataset of more than 1.6 million newborns across a period of over 10 years, with over 160,000 newborns screened annually. In this national programme only children receiving a hearing screening at the NICU were not included. Therefore, our study had high power, and we were able to identify that the optimal timing for OAE newborn hearing screening is between days five and thirteen after birth. However, our study also had some limitations. First, the main research question concerned the percentage of newborns who incorrectly did not pass the first test. We excluded all newborns referred to the Speech and Hearing Centre after three screening sessions, since diagnostic outcomes were not available in the dataset. As a result, newborns who were referred but diagnosed with normal hearing, i.e., false positive cases, were also excluded. This concerned approximately 400 unnecessarily excluded false positive newborns per year out of approximately 166,000 (±0.24%). Hence, the false positive rates were actually about 0.24% higher than presented. Second, the registration of hearing screening does not contain the times of birth and screening. Therefore, an analysis of hours from birth was not possible. Due to dates being analysed without times, a difference of one day may represent a time span as short as a few minutes or as long as almost 48 h. Third, it was not possible to determine to what extent and for which newborns there were errors in the registration of the dates. The registration of prematurely born newborns was incomplete. Prematurity was only recorded if the screening took place fourteen days or more after birth, and it was explicitly checked whether prematurity could explain the delayed screening. Since this information was unavailable for the remaining newborns, it is likely that not all premature newborns were excluded from the analyses. Fourth, several factors were likely to influence the results with regards to our second research question. We took into account the PCHC organization, since the probability of a referral differed between the organizations. Another factor likely influencing the probability of a referral is the individual screener conducting the test. Although screener-level data were not available for this study, it is plausible that some screeners achieve better outcomes than others, and that their personal experience with the ESIII device may also affect referral rates. It was not possible to include these data in the analyses.

## 5. Conclusions

This study demonstrated that the timing of the first OAE hearing screening significantly impacts the false positive referral rate on this test and should be considered in neonatal hearing screening programme implementation. Screening between days five and thirteen showed the lowest false positive referral rates. Additionally, it appeared that the likelihood of a false positive first OAE test was significantly higher when using the ESIII compared to the ESI/II, even if experience with using the ESIII had been acquired in the previous year. Thus, we advise monitoring false positive rates and other test outcomes in daily practice before introducing new equipment on a large scale in a screening programme.

## Figures and Tables

**Table 1:** Referral rate per year, nationwide and separately by device type (*N* = 323,194).

	% Referrals Nationwide	% Referrals with ESI/II	% Referrals with ESIII
2022	5.1%	4.4%	6.0%
2023	5.4%	4.6%	6.0%

ES = Echoscreen.

**Table 2:** Relationship between type of OAE screening device and referral rate during first screening (*N* = 159,912).

	Odds Ratio (OR)	95% Confidence Interval (CI)		*p*-Value
		Lower	Upper	
Intercept	0.038	0.033	0.044	<0.001
Device: ESI/II (ref)	^1^			
ESIII	1.844	1.650	2.061	<0.001
Age: Day 5 through 13 (ref)	^1^			
Till day 4	1.276	1.168	1.395	<0.001
≥Day 14	2.047	1.832	2.286	<0.001
PCHC type: Screening at home (ref)	^1^			
Screening at well-baby clinic	1.047	0.850	1.291	0.646
Experience: %ESIII in 2022	1.085	0.810	1.454	0.589
Experience * [device = ESI/II] (ref)	^1^			
Experience * [device = ESIII]	0.626	0.492	0.797	<0.001
Age (till day 4) * [device = ESIII]	0.931	0.836	1.036	0.187
Age (≥day 14) * [device = ESIII]	0.829	0.722	0.951	0.008

Note: Multilevel logistic regression model with referral rate in 2023 at first OAE screening as dependent variable and OAE device, age at first screening, type of preventive child healthcare (PCHC) organization, experience with ESIII in previous year, and interaction terms as independent variables. OAE = otoacoustic emission; ES = Echoscreen; %ESIII in 2022 = use of ESIII in 2022 within each PCHC organization (as percentage of all first OAE screenings), as measure of experience with ESIII; ^1^ reference group; OR is 1.

**Table 3:** Referral rates per age group and device type according to the multilevel logistic regression model (*N* = 159,912).

Age	ESIII	ESI/II	Ratio ESIII/ESI/II per Age Group
≥Day 14	11.8%	7.7%	1.53
Till day 4	8.3%	4.8%	1.72
Day 5 through 13	7.0%	3.8%	1.84

Note: These referral rates are the adjusted probabilities at baseline values (screening by heel prick organizations, with 0% use of ESIII in 2022). ES = Echoscreen.

## Data Availability

The data that support the findings of this study are available from the NSDSK, but restrictions apply to the availability of these data. Therefore, data are not publicly available. Requests to access the datasets should be directed to the NSDSK: nsdsk@nsdsk.nl.

## Abbreviations

The following abbreviations are used in this manuscript:

AABR	automated auditory brainstem response
CI	confidence interval
ES	Echoscreen
NICU	neonatal intensive care unit
NSDSK	the Dutch Foundation for the Deaf and Hard of Hearing Child
OAE	otoacoustic emission
OR	odds ratio
PCHC	preventive child healthcare
Ref	reference group
TNO	Netherlands Organisation for Applied Scientific Research
